# MTHFR as a Novel Candidate Marker for Litter Size in Rabbits

**DOI:** 10.3390/ani14131930

**Published:** 2024-06-29

**Authors:** Jie Yang, Zhiyuan Bao, Jiali Li, Tingting Lu, Jiawei Cai, Shaoning Sun, Ning Shen, Yang Chen, Bohao Zhao, Xinsheng Wu

**Affiliations:** 1 College of Animal Science and Technology, Yangzhou University, Yangzhou 225009, China; 19851563850@163.com (J.Y.); 18352764997@163.com (Z.B.); li1193117036@163.com (J.L.); yzultt@163.com (T.L.); cjwcjw520800@163.com (J.C.); 19952323987@163.com (S.S.); s2022_sn@163.com (N.S.); yangc@yzu.edu.cn (Y.C.); 2 Joint International Research Laboratory of Agriculture & Agri-Product Safety, Yangzhou University, Yangzhou 225009, China

**Keywords:** rabbits, MTHFR, SNPs, litter size, ovarian granulosa cells

## Abstract

**Simple Summary:**

Litter size is a crucial trait in animal reproduction. This study investigated the expression of the follicle-related gene methylenetetrahydrofolate reductase (*MTHFR*) in high- and low-fertility groups, together with the effects of the *MTHFR* expression level on ovarian granulosa cells and its association with litter size. The results indicated that the overexpression of *MTHFR* promoted apoptosis and inhibited proliferation in granulosa cells, while regulating the expression of follicle-related genes. A significant association was also found between both the total and alive litter sizes and the CC genotype with the variant g.-680C>A in the core promoter region of *MTHFR*. These findings suggest that the CC genotype could serve as a useful breeding marker in rabbits.

**Abstract:**

Litter size is a significant economic trait during animal reproduction. This current study attempted to decipher whether *MTHFR* promotes the apoptosis of granulosa cells (GCs) and inhibits their proliferation by investigating the effects of the *MTHFR* gene using flow cytometry and a Cell Counting Kit-8 (CCK-8) assay. *MTHFR* is linked with ovarian follicle development in the reproductive performance of 104 female New Zealand rabbits. We observed that *MTHFR* could regulate the mRNA of follicular development-related genes (*TIMP1*, *CITED1*, *FSHR*, *GHR*, *HSD17B1*, and *STAR*) with a qRT-PCR, and we observed the protein expression of CITED1 and GHR using a western blot (WB) analysis. The dual luciferase activity assays helped identify the core promoter region of the *MTHFR* gene, and the polymorphism of the *MTHFR* promoter region was studied using Sanger sequencing. The results indicated four single nucleotide polymorphisms (SNPs) within the core promoter region, among which the g.-680C>A locus was significantly associated with both the total and alive litter sizes. Additionally, the CC genotype was associated with the largest total and alive litter sizes, compared to the CA and AA genotypes (*p* < 0.05). In conclusion, this study investigated the effects of *MTHFR* on ovarian granulosa cells and its association with selected reproductive parameters in rabbits. The results provide a theoretical foundation for the use of *MTHFR* as a molecular marker in rabbits.

## 1. Introduction

The ovary is a vital reproductive organ in female animals, developing oocytes and sex hormones [1]. The primary functional cells of the ovary include ovarian GCs, whose proliferation and differentiation are vital in follicular growth and development. Litter size is a significant economic trait during animal reproduction, becoming a crucial indicator of livestock efficiency. Litter size is closely associated with ovarian follicular development, where heightened follicle-stimulating hormone and estrogen levels increase the number of ovulated oocytes [2], improving animal litter sizes. Several studies have depicted the link between follicular development genes and litter size. The SNP loci associated with the prolactin receptor (*PRLR*) gene, which is involved in the animal reproductive function, are closely linked with the dominant genotypes that enhance sow farrowing traits [3]. Bone morphogenetic protein 15 (BMP15) and growth differentiation factor 9 (GDF9), secreted by ovarian follicles, participate in ovulation, steroidogenesis, and corpus luteum formation [4,5]. To enhance the livestock litter size, further investigations are required into the genes connected with ovarian follicle development.

In our previous study, several genes linked with follicular development, steroid hormone synthesis, and ovulation were screened using proteomic sequencing in high and low fecundity groups. Among them, the *MTHFR* gene was associated with follicular development. The Proteomic-Seq dataset identifier number was PRJNA888836. MTHFR, as a critical enzyme in the methionine folate metabolism system, can restore 5,10-methylenetetrahydrofolate to 5-methylenetetrahydrofolate, the primary form of the folate metabolism cycle [6]. The folate metabolism may affect estradiol synthesis and other ovarian steroids [7]. MTHFR can provide methyl to synthesize purines and pyrimidines, followed by DNA, RNA, and protein methylation in vivo. It also maintains a normal homocysteine level in vivo, with a critical role in follicular and embryonic development [8]. Currently, *MTHFR* research mainly focuses on studying different diseases and polymorphisms in human beings. The *MTHFR* exon rs915014 locus has been associated with an elevated risk of atherosclerosis [9]. Additionally, the *MTHFR* C677T polymorphism has correlated with maternal Down syndrome and a heightened stroke risk [10,11]. The *MTHFR* gene has been studied in animal reproduction. Embryonic MTHFR is essential in the normal development of blastocysts and can improve the care of early embryos by controlling folate-related nutritional conditions in vivo and in vitro [12]. Furthermore, MTHFR is expressed inside the testes of mammals and directly affects spermatozoa, with a significantly larger expression among adult yaks than juveniles [13].

Polymorphism studies of the *MTHFR* gene related to the reproductive performance of animals have rarely been reported. This current research investigates the proliferation and apoptosis of *MTHFR* in rabbit ovarian GCs, while detecting *MTHFR* and the genes associated with follicular development, by analyzing mRNA and protein levels. Meanwhile, this study investigated the correlation between SNPs in the promoter region of the *MTHFR* gene and the reproductive traits of rabbits to screen out the genotypes with large litter sizes. The aim of this study was to investigate the regulation of ovarian granulosa cells by *MTHFR* and the relationships between its genetic polymorphisms and selected parameters of reproductive performance in order to identify a molecular marker for reproductive performance in rabbits. The findings could provide a necessary theoretical basis and economic benefits for the genetic improvement of rabbit reproductive performance.

## 2. Materials and Methods

### 2.1. Animal and Sample Collection

Ear sample tissues were obtained from the same batch of 104 New Zealand female rabbits (age: 6 months) reared in the same environment (temperature, water, diet, and breeding management). The rabbit gestation period is about 1 month, and the number of rabbits per litter was recorded manually within 12 h of parturition, for an average of three consecutive litters per female. The female rabbits selected for this study were of a similar health and body condition, received food and water ad libitum in an appropriate environment, and lactated for approximately 35 days. All of the experimental females in this study had grandparents from at least 30 family lines that were not related to each other. The rabbit wools were removed from the collected ear tissues using ophthalmic scissors. They were put inside 1.5 mL centrifuge tubes filled beforehand with 75% ethanol and stored at −20 °C, before the DNA was extracted using a simple extraction kit (Tiangen, Beijing, China).

### 2.2. Isolation and Culture of GCs

The ovarian tissue samples were collected from the rabbits, placed in a precooled PBS buffer (Biosharp, Beijing, China), and washed at least thrice. The tissues were transferred onto Petri dishes, after which the follicles were punctured with a 1 mL syringe needle. Subsequently, the GCs were separated. The supernatants were filtered using a 200-mesh cell sieve and centrifuged to eliminate the supernatants. Next, 2 mL of red blood cell lysis buffer was added, and the supernatant was centrifuged after blowing and mixing. Then, the cells were resuspended using the DMEM-F12 medium (DMEM/F12; Gibco^®^, Grand Island, NY, USA), 10% fetal bovine serum (Gibco^®^, Grand Island, NY, USA), and 1% double antibody (a penicillin–streptomycin solution). Finally, the cells were inoculated into six-well plates and cultured at 37 °C inside a 5% CO_2_ incubator. There is some additional literature on the isolation and culture of GCs [14].

### 2.3. MTHFR Cloning and Vector Construction

The pcDNA3.1(+) plasmid was a vector, with *HindIII* and *EcoRⅠ* being the chosen digestion sites. Beijing Tsingke Biotech Co, Ltd. (Beijing, China) helped design the one-step cloning primers (Table S1), while a Phanta Max High Fidelity Enzyme (Vazyme, Nanjing, China) was utilized to amplify the PCR product. A MiniBEST Agarose Gel DNA Extraction Kit V.4.0 (Takara, Dalian, China) was used for the purification. The coding sequence amplification product of MTHFR was linked with the bis-enzymatic digested pcDNA3.1(+) vector and transferred into *E. coli* DH5α receptor cells (Takara, Dalian, China). Suzhou GenePharma Co. Ltd. (Suzhou, China) helped design and purchase the siRNA (Table S1) to transfect the GCs depending on the rabbit MTHFR (NCBI Reference Sequence: XM051834356.1).

### 2.4. Quantitative Real-Time PCR (qRT-PCR)

The RNA was extracted from the transfected cells with a Total RNA Extraction Kit (Tiangen, Beijing, China), and the OD value was measured using a Nanodrop-2000 spectrophotometer (Thermo Electron Corporation, Waltham, MA, USA) for the quantification of the RNA concentration and the integrity detection. HiScript III RT SuperMix for qRT (+gDNA wiper) (Vazyme, Nanjing, China) helped reverse-transcribe the RNA samples for the cDNA production. This was quantified using the ChamQ SYBR qPCR Master Mix (Vazyme, Nanjing, China) in the qRT-PCR. The RNA levels were determined using the 2^−ΔΔCT^ method, with GAPDH as an internal reference. All the procedures followed the manufacturer’s instructions, and the specific primers are provided in Table S2.

### 2.5. Cell Proliferation and Apoptosis Assays

The cells were transfected using Lipofectamine™ 2000 (Invitrogen, Waltham, MA, USA) and then inoculated into 96-well plates, before being incubated at 37 °C in a 5% CO_2_ incubator. The samples were incubated with 10 μL of CCK-8 reagent (Cell Counting Kit-8, Beyotime, Shanghai, China) for 1 h. Their absorbance was measured at 450 nm with an enzyme marker (Tecan, Männedorf, Switzerland) after 0, 24, 48, and 72 h. Apoptosis was detected in the GCs with the Annexin V-FITC/PI Apoptosis Detection Kit (Vazyme, Nanjing, China). The samples were examined using flow cytometry (CytoFLEX S, Beckman Coulter, Brea, CA, USA) within 1 h of staining and subsequently analyzed with the CytExpert 2.3 (Beckman Coulter, Brea, CA, USA) software.

### 2.6. Western Blotting

The protein was extracted from the transfected cells using RIPA lysate (PPLYGEN, Beijing, China), and the protein concentration was measured with the BCA Protein Assay Kit (Beyotime, Shanghai, China). Equal protein amounts were applied to the SDS-PAGE, transferred onto membranes, and blocked using 5% skimmed milk. After washing in 1× TBST, anti-CITED1 (1:2000; Proteintech, Cat#: 26999-1-AP), anti-MTHFR (1:10,000; Proteintech, Cat#: 66612-1-Ig), GHR (1:1000; Proteintech, Cat#: 20713-1-AP), and anti-GAPDH (1:100,000; Proteintech, Cat#: 60004-1-Ig) antibodies were incubated overnight at 4 °C with shaking. The secondary antibodies were incubated with anti-mouse (1:10,000; Proteintech, Cat#: SA00001-1) and anti-rabbit (1:10,000; Proteintech, Cat#: SA00001-2) antibodies for 1 h at room temperature and washed with 1× TBST wash. Finally, development was conducted on a TanonABL X5 series developing system (Tannen Technology, Shanghai, China).

### 2.7. Dual Luciferase Assay Analysis of MTHFR Promoter Region

The core promoter regions were predicted with the Promoter-2.0 online website (Promoter 2.0-DTU Health Tech-Bioinformatic Services). The promoters of each segment, such as P1 (+200 to −300), P2 (+200 to −800), P3 (+200 to −1400), and P4 (+200 to −2000), were cloned within the pGL3.0-Basic plasmid, becoming a linker vector. Human embryonic kidney 293T cells (HEK 293T cells) were co-transfected using a pRL-TK vector and the pGL3-basic vector, which served as a control. The Dual-Luciferase Reporter System (Promega, Madison, WI, USA) helped detect the relative luciferase expression level after 48 h of cell transfection. The detailed primer information is shown in Table S3.

### 2.8. Identification of MTHFR Promoter Polymorphisms

The sequence of the rabbit MTHFR gene was retrieved from NCBI, and the core promoter primer of the MTHFR gene was designed with the NCBI Primer-BLAST software (Primer designing tool (nih.gov)) (Table S3). Then, 104 extracted ear samples became templates of 1 µL each, with 25 µL of 2× Rapid Taq Master Mix, upstream and downstream primers of 2 µL each, and 20 µL of ddH_2_O, making a total reaction system of 50 µL. The PCR amplification procedure included pre-denaturation at 95 °C for 3 min, denaturation at 95 °C for 15 s, annealing at 61 °C for 15 s, and extension at 72 °C for 15 s. The cycles were repeated 35 times and extended at 72 °C for 5 min before storage at 4 °C.

### 2.9. Statistical Analysis

The statistical analysis was performed using the SPSS 25.0 software (SPSS Inc., Chicago, IL, USA). The statistical significance was calculated using the paired samples *t*-test and a one-way ANOVA. The graphs were plotted with GraphPad Prism8. Haploview 4.2 software helped to evaluate the linkage disequilibrium and to construct the haplotypes. All the experiments were performed with at least three biological replicates. The error bars in the results include the mean ± standard deviation (*p* < 0.05 significant difference; *p* > 0.05 non-significant difference).

## 3. Results

### 3.1. Overexpression and Knockdown of MTHFR in GCs

**Figure 1:**
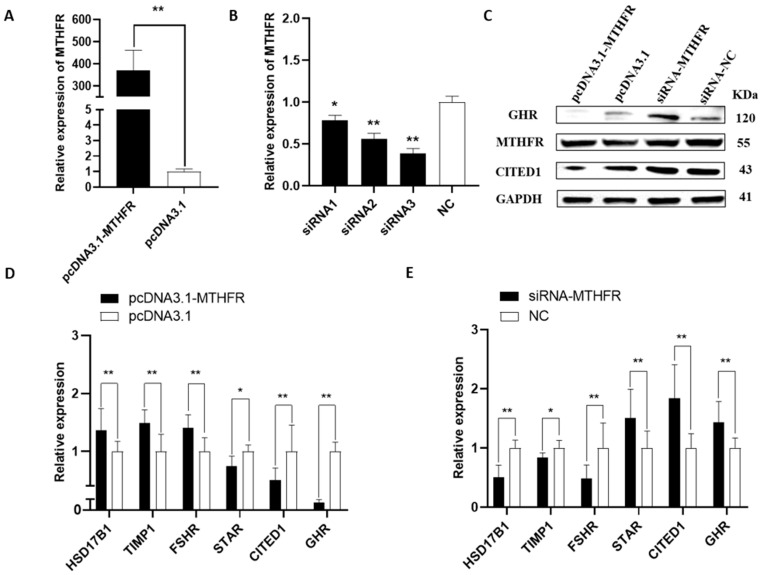
The effects of mRNA and protein on follicular development-related genes after the overexpression and knockdown of MTHFR in GCs: (**A**) pcDNA3.1-MTHFR significantly enhanced the MTHFR expression level; (**B**) siRNA-MTHFR significantly reduced the mRNA expression level of MTHFR; and (**C**–**E**) the protein and mRNA expression levels of follicular development-associated genes after the overexpression and knockdown of MTHFR, respectively. * *p* < 0.05, ** *p* < 0.01.

All *MTHFR* and related gene levels were examined in the rabbit GCs with a qRT-PCR and WB analysis. The effectiveness of the pcDNA3.1-MTHFR and siRNA-MTHFR was assessed in the rabbit GCs using a qRT-PCR. The results demonstrated that pcDNA3.1-MTHFR could significantly upregulate the *MTHFR* mRNA expression levels (*p* < 0.01, Figure 1A). Consequently, *MTHFR* overexpression could significantly upregulate the mRNA expression levels of follicular development-related genes, including *HSD17B1*, *TIMP1*, and *FSHR*, while downregulating the gene expression of *STAR*, *CITED1*, and *GHR* (*p* < 0.05, Figure 1D). Furthermore, *MTHFR* knockdown could decrease its mRNA expression (*p* < 0.05, Figure 1B) and downregulate the mRNA expression levels of the genes associated with follicular development, including *HSD17B1*, *TIMP1*, and *FSHR*. However, the knockdown significantly upregulated the gene expression of *STAR*, *CITED1*, and *GHR* (*p* < 0.05, Figure 1E). Additionally, *MTHFR* overexpression considerably increased its protein expression level while reducing the CITED1 and GHR levels (Figure 1C).

### 3.2. MTHFR Regulates the Cell Proliferation in GCs

**Figure 2:**
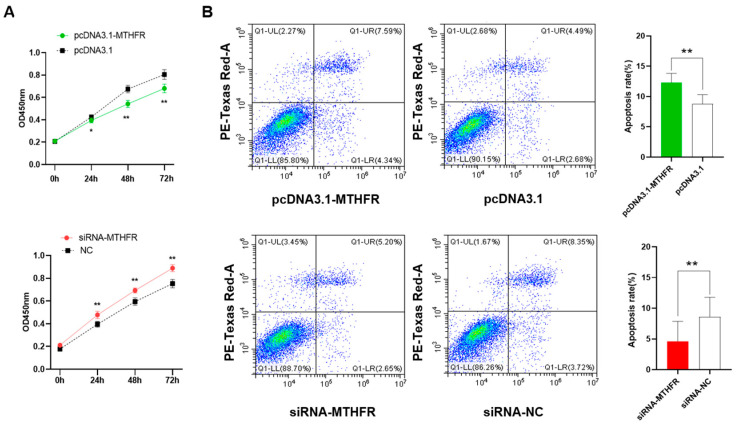
The overexpression and knockdown of MTHFR, which (**A**) significantly inhibited the GC proliferation and (**B**) significantly enhanced the apoptosis of GCs. * *p* < 0.05, ** *p* < 0.01.

The CCK-8 assay helped analyze the overexpression and knockdown of *MTHFR*, which modulates cell proliferation in GCs. The results showed that the overexpression of *MTHFR* could significantly inhibit the cell proliferation of GCs (*p* < 0.01, Figure 2A) and the knockdown of *MTHFR* promoted significant cell proliferation in GCs (*p* < 0.01). The flow cytometry analysis demonstrated that overexpressing *MTHFR* significantly promoted cell apoptosis, while *MTHFR* knockdown suppressed the cell apoptosis level in GCs (*p* < 0.01, Figure 2B).

### 3.3. Polymorphism in the MTHFR Gene Promoter Region

**Figure 3:**
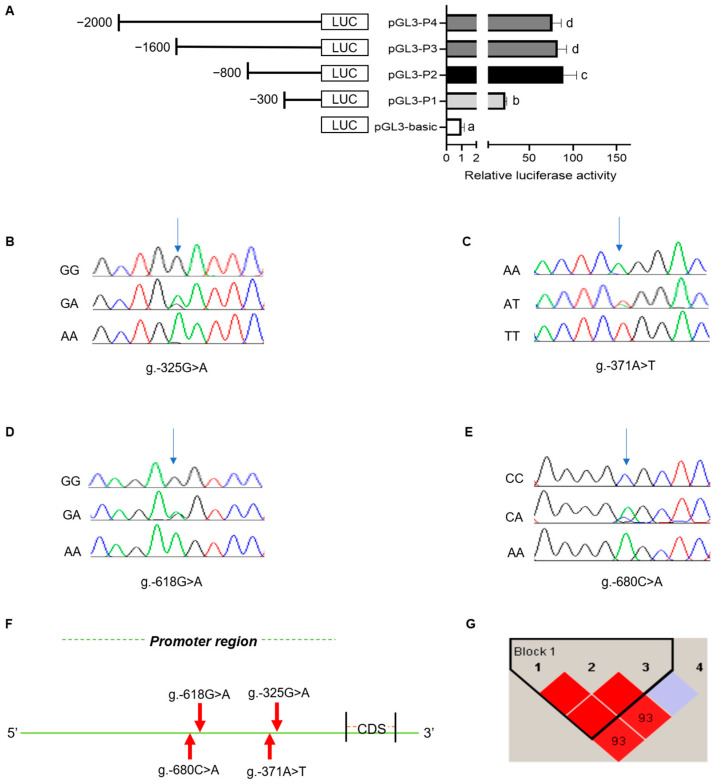
(**A**) Dual luciferase assay of the MTHFR core promoter indicating the highest dual luciferase levels from −300 to −800. The four SNPs were as follows: (**B**) g.-325G>A, (**C**) g.-371A>T, (**D**) g.-618G>A, and (**E**) g.-680C>A; and the different letters (abcd) depict varied significance, where *p* < 0.05. The colours of different peaks represent different bases, green for base A, red for base T, blue for base C and black for base G. (**F**) The SNP detection in the MTHFR promoter region. (**G**) The linkage disequilibrium analysis of the four SNPs in the MTHFR. The color in the haplotype blocks ranges from dark to light, depicting the degree of linkage from high to low. r^2^ represents the statistical association of the two loci.

The promoter sequence of the rabbit *MTHFR* gene was predicted using Promoter 2.0 software. The results indicated that the potential core promoter region of the *MTHFR* gene was located at −400 bp. The luciferase recombinant plasmid was constructed based on the promoter amplified fragment lengths. The luciferase activity was detected using the Dual-Luciferase Reporter System, with the highest luciferase activities between −300 and −800 in the promoter region of *MTHFR* (Figure 3A). Therefore, the core promoter region of *MTHFR* was located between −300 and −800.

Then, the polymorphisms of the *MTHFR* core promoter were investigated. The SNP analyses showed that there were four SNPs in the *MTHFR* core promoter, which were g.-325G>A, g.-371A>T, g.-618G>A, and g.-680C>A (Figure 3B–F). Moreover, the genotype and gene frequencies were analyzed among the New Zealand female rabbits for each SNP (Table 1). The χ^2^ test analysis indicated that all the SNPs followed the Hardy–Weinberg law (*p* > 0.05). The g.-618G>A locus demonstrated a low polymorphism level (*PIC* < 0.25) among the genetic loci analysis. In contrast, the remaining SNPs displayed a moderate polymorphism (0.25 < *PIC* < 0.5). The A was the dominant allele at the g.-325G>A locus, while T was at the g.-371A>T locus. GG and CC were the dominant allelic phenotypes at loci g.-618G>A and g.-680C>A, whereas G and C were the dominant alleles at loci g.-618G>A and g.-680C>A. A linkage disequilibrium (LD) analysis was performed on the four SNPs with the Haploview 4.2 software. This study helped construct a haplotype block with three haplotypes, H1(ATG), H2(GAG), and H3(GAA), with frequencies of 0.587, 0.268, and 0.144, respectively. A table showing the *MTHFR* gene haplotypes, diplotype frequency, and an analysis of their association with selected parameters of reproductive performance is provided in Supplementary File S2. The above three haplotypes were combined to produce four haplotype combinations: H1H1, H1H2, H1H3, and H2H3, with H1H1 being the most frequent combination. The combination of g.-325G>A and g.-371A>T presents in a high LD (r^2^ = 1) and that of g.-325G>A and g.-680 C>A presents in a low LD (r^2^ = 0.478).

### 3.4. Association Analysis of the SNP Loci in the MTHFR Promoter Region with Selected Reproductive Parameters

The loci of the SNPs that conformed to the Hardy–Weinberg law were analyzed to associate with selected reproductive parameters in New Zealand female rabbits. No significant differences were observed among the four SNPs in relation to the average weight at birth, survival rate at birth, average weight of 3- and 5-week-old kids, and the average weight of weaned female rabbits at 5 weeks (*p* > 0.05). Only the g.-680C>A locus was significantly linked to both the total and alive litter sizes (*p* < 0.05). Furthermore, the total and alive litter sizes of the CC genotype within the g.-680C>A locus were higher than the CA and AA genotypes (Supplementary File S1). However, no significant differences could be observed when linking the four haplotype combinations with the selected reproductive parameters. Therefore, the CC genotype could be a genetic marker, and choosing female rabbits with this genotype for breeding could increase the litter size.

## 4. Discussion

Reproductive traits are economically essential indicators of production and development in livestock production. Thus, the higher the fertility of an animal, the lower the production costs with higher economic breeding benefits. The litter size of rabbits is a vital production performance trait that affects the economic benefits of the rabbit industry.

Ovarian follicular development is the foundation of female reproduction. This current article deciphered the effect of the follicular development gene *MTHFR* on rabbit ovarian GCs. MTHFR is a folate-reducing enzyme linked with ovarian follicular activity [15]. The overexpression and knockdown of *MTHFR* could suppress GC proliferation and enhance its apoptosis. Various hormones synthesized by the steroidogenic pathway in the ovary are critical in regulating the reproductive function while maintaining fertility using specific nuclear receptors [16]. Human uterine and ovarian GCs express the 17β-hydroxysteroid dehydrogenase type 1 (*HSD17B1*) gene [17], while knocking down the *HSD17B1* gene in mice ovaries has altered ovarian steroid hormones, decreased the number of follicles, and reduced fertility [18]. Steroid hormone synthesis is regulated by STAR [19], expressed in the ovaries of different animals, and is intimately associated with follicular development [20]. One study depicted a notable elevation in the steroidogenic acute regulatory protein (*STAR*) gene expression in the pituitary glands of polytocous sheep compared to monotocous sheep [21]. CBP/P-300 interacting transactivator 1 (CITED1) is a transcriptional cofactor crucial to the normal development of embryos and trophoblast cell survival during the embryonic stages [22]. It achieves transcriptional activation by improving the DNA-binding transcription factor SMAD and facilitates estrogen receptor signaling to control estrogen-dependent activation [23,24]. In this study, *MTHFR* could upregulate *HSD17B1*, *TIMP1*, and *FSHR* and downregulate *STAR*, *CITED1*, and *GHR*. MTHFR is crucial in the cyclic folate metabolism and rapid cell growth in follicular and embryonic development. While researching the effect of *MTHFR* on polymorphism, patients carrying the mutant allele (rs1801133) have higher follicle-stimulating hormone (FSH) levels, requiring higher FSH doses to enhance follicular development. Follicle-stimulating hormone receptor (FSHR) mediates and specifically binds FSH, activating the intracellular signaling pathway with a crucial role in the reproductive process [25]. Additionally, several SNPs are present inside the 5‘UTR of the *FSHR* gene in sheep, significantly associated with the distribution of sheep breeds and litter sizes [26]. The growth hormone receptor (GHR) receptor for the growth hormone (GH) is expressed inside human ovaries [27]. The knockdown of the gene resulted in a reduced ovulation rate and litter size within mice [28]. The tissue inhibitor of metalloproteinases-1 (*TIMP1*) gene is highly expressed in GCs, which increases the transcript levels of some genes associated with lambing, while regulating the physiological cycle of mice at the uterine and ovarian levels [29,30]. It has been suggested that *TIMP1* may be a candidate gene for embryo implantation and survival [31]. Subsequent investigation and exploration of the genetic *MTHFR* regulation is necessary. *MTHFR* could inhibit cell proliferation and enhance the apoptosis of GCs, suggesting that *MTHFR* may control follicular development by affecting ovarian GC activities.

Several SNPs’ loci are related to litter size, including the G protein-coupled receptor 54 (GPR54) [32], estrogen receptor (ESR) gene [33], progesterone receptor (PGR) [34], follicle-stimulating hormone (FSHβ) [35], and oviductal glycoprotein 1 (OVGP1) [36]. These genes have been associated with reproductive traits in rabbits and are useful for the selection of superior genotypes for breeding, as well as for the identification of molecular markers. However, the *MTHFR* genetic polymorphisms studied in this paper primarily focused on the two loci of C677T and A1298C in various diseases [37,38]. Thus, fewer studies were linked with the reproductive performance of animals. Four SNPs were detected in the *MTHFR* core promoter region using the dual luciferase assay, all following the Hardy–Weinberg law (*p* > 0.05). The analysis of the selected reproductive parameters of New Zealand female rabbits revealed a significant difference in the g.-680C>A locus, in which the CC genotype was higher than the total and alive litter sizes of CA and AA. Therefore, the CC genotype could be the dominant genotype. As the promoter is a crucial gene regulatory region, it can be linked with initiating and regulating the gene transcription by binding to specific transcription factors [39]. Therefore, base mutations within the gene promoters could alter the recognition and binding of the transcription factors, leading to apparent changes in the luciferase activity while affecting the genetic transcriptional expression. Polymorphisms in the *MTHFR* gene promoter may regulate its transcription and are connected with litter size traits. However, further investigation is required. The LD analysis of the four SNPs inside the core promoter region of *MTHFR* depicted that the combinations of g.-325G>A and g.-371A>T and g.-325G>A and g.-680C>A had r^2^ values more significant than one-third in the LD. Values of r^2^ that are greater than one-third indicate a close linkage of the two SNP loci [40]. This suggests that these two loci can be inherited. However, the association of the g.-325G>A and g.-371A>T loci with selected reproductive parameters was insignificant. No significant differences between the association of the selected reproductive parameters and any of the four *MTHFR* gene haplotype combinations could be observed. A larger sample size may be required for additional validation.

## 5. Conclusions

In conclusion, the *MTHFR* gene was successfully cloned from rabbits. Furthermore, the overexpression and knockdown of *MTHFR* could inhibit cell proliferation, promote GC apoptosis, and regulate the genes associated with follicular development. Among the four SNPs in the core promoter region, the LD was combined with g.-325G>A, g.-371A>T, g.-325G>A, and g.-680C>A. However, only the g.-680C>A locus notably impacted both the total and alive litter sizes. Thus, the values for the total and alive litter sizes were highest in the CC genotype female rabbits. To date, there have been few studies on reproductive traits such as litter size in rabbits at the molecular genetic level. The results of this present study highlight the importance of *MTHFR* in the regulation of rabbit ovarian granulosa cells, as well as litter sizes. This study provides an essential theoretical basis for the additional investigation of the *MTHFR* gene in rabbits to enhance litter sizes among New Zealand female rabbits. It also has specific implications for using molecular marker-assisted technology and improving reproductive performance among female rabbits. In future work, we will continue to verify the use of *MTHFR* SNPs as biomarkers in a larger rabbit population. The *MTHFR* gene needs to be investigated further in different breeds of rabbits or other species.

## Figures and Tables

**Table 1:** Population genetic information of the four SNPs of the MTHFR gene in female rabbits. *n* = 104.

SNPs	Genotype Frequency		Allele Frequency		He	χ^2^	PIC	*p*-Value
	GG	0.21 (22)	G	0.41				
g.-325G>A	GA	0.40 (42)	A	0.59	0.485	2.913	0.367	0.233
	AA	0.39 (40)						
	AA	0.21 (22)	A	0.41				
g.-371A>T	AT	0.40 (42)	T	0.59	0.485	2.913	0.367	0.233
	TT	0.39 (40)						
	GG	0.73 (76)	G	0.86				
g.-618G>A	GA	0.25 (26)	A	0.14	0.247	0.017	0.216	0.992
	AA	0.02 (2)						
	CC	0.54 (56)	C	0.72				
g.-680C>A	CA	0.36 (38)	A	0.28	0.402	0.871	0.321	0.647
	AA	0.10 (10)						

Note: He: expected heterozygosity; χ^2^: chi-square value; and PIC: polymorphism information content.

## Data Availability

The raw data supporting the conclusions of this article will be made available by the authors on request.

## Supplementary Materials

The following supporting information can be downloaded at https://www.mdpi.com/article/10.3390/ani14131930/s1. Figure S1: western blot figure for CITED1; Figure S2: western blot figure for MTHFR; Figure S3: western blot figure for GHR; Figure S4: western blot figure for GAPDH; Table S1: the sequence primers that overexpress MTHFR and siRNA; Table S2: information on the primers used for the qRT-PCR; Table S3: the primers used for constructing the luciferase reporter vector; Supplementary File S1: the association analysis of the MTHFR gene polymorphisms, showing the selected reproductive parameters; and Supplementary File S2: the analysis of the association among the MTHFR gene haplotypes, diplotype frequency, and selected reproductive parameters.
