# Supplementary figures and images for: MTHFR as a Novel Candidate Marker for Litter Size in Rabbits
Source: Animals (Basel). 2024 Jun 29;14(13):1930. doi: 10.3390/ani14131930 (PMC11240429; doi:10.3390/ani14131930)

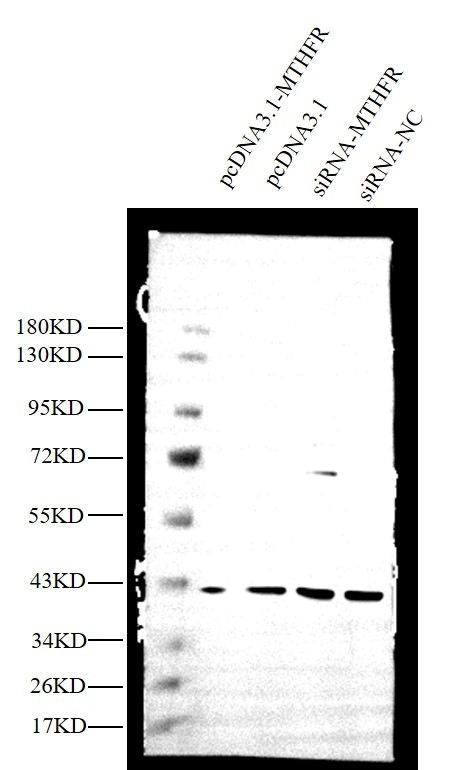

Supplement: Supplementary file 1 [file animals-14-01930-s001.zip › Figure S1. Western Blot Figure for CITED1.jpg]

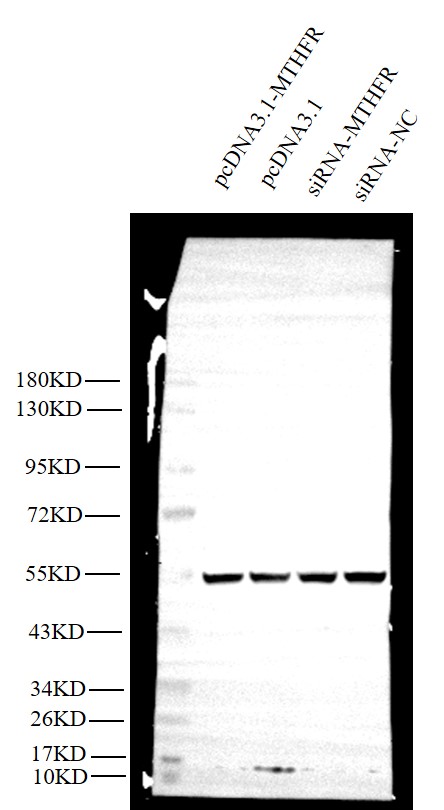

Supplement: Supplementary file 1 [file animals-14-01930-s001.zip › Figure S2. Western Blot Figure for MTHFR.jpg]

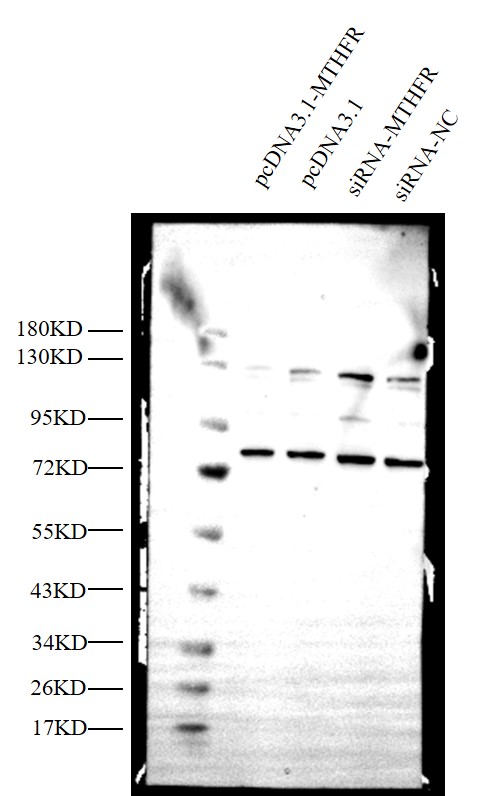

Supplement: Supplementary file 1 [file animals-14-01930-s001.zip › Figure S3. Western Blot Figure for GHR.jpg]

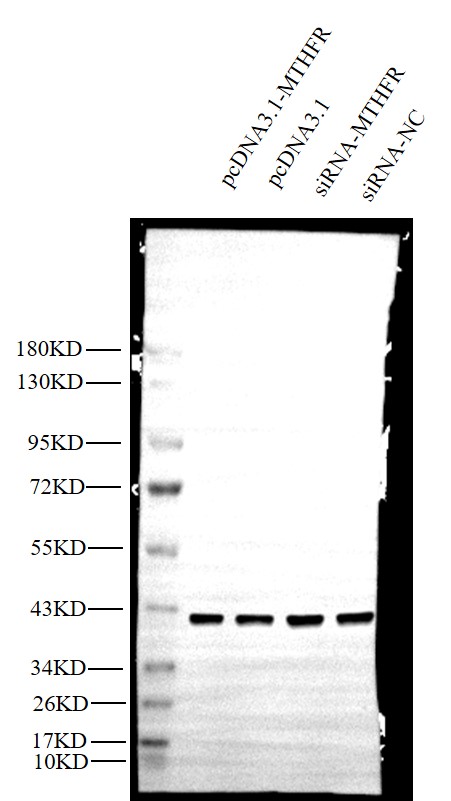

Supplement: Supplementary file 1 [file animals-14-01930-s001.zip › Figure S4. Western Blot Figure for GAPDH.jpg]
